# Fundoscopy-directed genetic testing to re-evaluate negative whole exome sequencing results

**DOI:** 10.1186/s13023-020-1312-1

**Published:** 2020-01-30

**Authors:** Ahra Cho, Jose Ronaldo Lima de Carvalho, Akemi J. Tanaka, Ruben Jauregui, Sarah R. Levi, Alexander G. Bassuk, Vinit B. Mahajan, Stephen H. Tsang

**Affiliations:** 1 Department of Ophthalmology, Columbia University, New York, NY USA; 2 Institute of Human Nutrition, Vagelos College of Physicians and Surgeons, Columbia University, New York, NY USA; 3 Jonas Children’s Vision Care and Bernard & Shirlee Brown Glaucoma Laboratory, New York-Presbyterian Hospital, Edward S. Harkness Eye Institute, New York, NY USA; 4 Department of Ophthalmology, Empresa Brasileira de Servicos Hospitalares (EBSERH) – Hospital das Clinicas de Pernambuco (HCPE), Federal University of Pernambuco (UFPE), Recife, Brazil; 5 Department of Ophthalmology, Federal University of São Paulo (UNIFESP), São Paulo, Brazil; 6 Department of Pathology & Cell Biology, and Columbia Stem Cell Initiative, Columbia University Medical Center, New York, NY USA; 7 Weill Cornell Medical College, New York, NY USA; 8 Department of Pediatrics, University of Iowa, Iowa City, IA USA; 9 Department of Ophthalmology, Byers Eye Institute, Stanford University, Palo Alto, CA USA; 10 Veterans Affairs Palo Alto Health Care Systems, Palo Alto, CA USA

**Keywords:** Whole exome sequencing, Inherited retinal diseases, Fundoscopy, Gene therapy, Whole genome sequencing

## Abstract

**Background:**

Whole exome sequencing (WES) allows for an unbiased search of the genetic cause of a disease. Employing it as a first-tier genetic testing can be favored due to the associated lower incremental cost per diagnosis compared to when using it later in the diagnostic pathway. However, there are technical limitations of WES that can lead to inaccurate negative variant callings. Our study presents these limitations through a re-evaluation of negative WES results using subsequent tests primarily driven by fundoscopic findings. These tests included targeted gene testing, inherited retinal gene panels, whole genome sequencing (WGS), and array comparative genomic hybridization.

**Results:**

Subsequent genetic testing guided by fundoscopy findings identified the following variant types causing retinitis pigmentosa that were not detected by WES: frameshift deletion and nonsense variants in the *RPGR* gene, 353-bp Alu repeat insertions in the *MAK* gene, and large exonic deletion variants in the *EYS* and *PRPF31* genes. Deep intronic variants in the *ABCA4* gene causing Stargardt disease and the *GUCY2D* gene causing Leber congenital amaurosis were also identified.

**Conclusions:**

Negative WES analyses inconsistent with the phenotype should raise clinical suspicion. Subsequent genetic testing may detect genetic variants missed by WES and can make patients eligible for gene replacement therapy and upcoming clinical trials. When phenotypic findings support a genetic etiology, negative WES results should be followed by targeted gene sequencing, array based approach or whole genome sequencing.

## Background

Inherited retinal diseases (IRDs) are observed in highly variable phenotypes in 1 in 2000 people [1]. To date, more than 250 IRD-causing genes have been identified [2]. The opsin 1 (medium- and long-wave-sensitive) and rhodopsin genes were the first to be discovered, identified in 8% of Caucasian males with red-green color blindness and 25% of autosomal dominant cases of retinitis pigmentosa, respectively [3–5]. The genomic era unfolded with the completion of the Human Genome Project in 2003 [6], which facilitated candidate gene analysis for the identification of causal genes in chromosomal locations determined through linkage analysis [7]. Successful identification of genetic changes in patients with clinical presentations of IRDs has driven the application of precision medicine for disease management and treatment. Therapeutic options such as adeno-associated virus vector-based gene therapy hold a great promise to reverse visual impairment in patients with IRDs [8, 9].

In contrast to dideoxy sequencing, next generation sequencing (NGS) has reduced the time it takes to sequence massive amounts of DNA from decades to months. Whole exome sequencing (WES) selectively targets the 20,000 coding genes that constitute approximately 2% of the human genome, as they are predicted to be responsible for 85% of rare and common inherited diseases [10]. However, genome-wide association studies (GWAS) have revealed that a significant proportion of variants within the noncoding genome are clinically relevant; mutations in the regulatory DNA sequences are either pathogenic themselves or they affect complex interactions between individual genetic features that lead to disease [11]. Such findings accentuate the inherent limitation of WES, as its coverage of exons and immediately adjacent introns consequently fails to identify variants in the remaining 98% of the genome. In addition to restricting the scope of sequencing, genetic structures such as high GC-percent regions, homopolymeric repeats and insertion or deletions (indels) greater than 20 to 50 nucleotides, are associated with increased rates in the failure of WES variant calling [12]. Copy number variations (CNVs) within an exon are covered by WES chemistry but likely to be missed in the reporting when the size exceeds 50 bp based on the analysis pipeline. For WES to detect structural genomic DNA arrangements and large CNVs, the variant analysis pipeline should be accompanied with array comparative genomic hybridization (CGH) analysis. Variant calling by WES is also limited to the scope of reported pathogenic gene variants, which opens the possibility of the association of the phenotype with a gene not previously associated with disease. Therefore, when clinical indications are prominent, a negative WES analysis should be re-evaluated, as it can be insufficient to exclude disorders in the differential diagnoses [13].

In this study, we present individuals and their family members in whom no disease-causing variants had been identified by clinical exome sequencing. Pathogenic or likely pathogenic variants were subsequently identified by targeted single-gene sequencing, gene panels, whole genome sequencing (WGS), or array CGH analysis, which provided genetic diagnoses of retinitis pigmentosa (X-linked RP) (MIM 300455), (RP62) (MIM 614181), (RP25) (MIM 602772), (RP11) (MIM 600138), Stargardt disease 1 (STGD1) (MIM 248200), and Leber congenital amaurosis 1 (LCA1) (MIM 204000). Through our investigation, we propose possible molecular mechanisms underlying the missed variant calls and emphasize the need for continued search for the causative variant in such cases. Furthermore, we suggest increased utilization of WGS, a more comprehensive type of NGS that has recently shown a significant reduction in cost [14].

## Subjects and methods

### Subjects

This study was approved by the Institutional Review Board of Columbia University Irving Medical Center and adhered to the tenets of the Declaration of Helsinki. Written informed consent was obtained from all participants per protocol. All clinical data, genetic information and imaging presented in this study are not identifiable to individual participant and are in accordance with HIPAA. The patients were referred to the Edward S. Harkness Eye Institute for genetic diagnosis following retinal evaluation. The molecular genetic reports of 638 participants seen over a 6-year period were screened. The selection criteria included all participants clinically diagnosed with IRDs whose genetic characterization was not identified by WES but was later detected through alternative genetic testing platforms.

### Clinical assessment

Clinical assessment of probands and family members included family history and a complete ophthalmic examination including visual acuity assessment, full-field electroretinogram (ffERG), indirect ophthalmoscopy, and retinal imaging performed following pupillary dilation. Color fundus photography, infrared reflectance imaging, spectral-domain optical coherence tomography (SD-OCT), and short-wavelength fundus autofluorescence (SW-AF, 488 nm excitation), were obtained using Spectralis HRA + OCT device (Heidelberg Engineering, Heidelberg, Germany). Wide-angle color fundus photography was performed using Daytona Optos device (Optos, Dunfermline, UK).

### Sequencing and variant pathogenicity analysis

DNA was isolated from peripheral whole blood of each participant for WES at the Personalized Genomic Medicine Laboratory at Columbia University Irving Medical Center. WES was performed as first-tier genetic testing for the unbiased search for the genetic cause of disease. WES was performed with Agilent SureSelectXT Human All Exon V5 + UTRs capture (Agilent Technologies Inc., Santa Clara, CA, USA) and Illumina HiSeq2500 sequencing technology (Illumina, San Diego, CA, USA). The WES output reads were mapped against the reference genome (GRCh 37/hg19) using NextGENe software (Softgenetics, State College, PA, USA) and our own proprietary analytical pipeline to sequence alignment for variant calling. Due to the technical limitations of sequence capture employed in this test, intronic variants were not predicted to be identified. Targeted sequencing of the *RPGR* gene was evaluated using long range PCR followed by DNA fragmentation and long read (250 bp-paired end) high-depth Illumina sequencing.

The following molecular diagnostic tests were ordered based on the patient’s family history and the clinical features: targeted gene sequencing and inherited retinal dystrophy panels due to the 100% exon coverage and 99% sensitivity for nucleotide base alterations as well as small deletions and insertions, WGS for the detection of noncoding variants, and array CGH of IRD genes for the detection of structural variants such as CNVs with 99% sensitivity for the detection of nucleotide base changes. Gene sequencing was conducted at the Personalized Genomic Medicine Laboratory at Columbia University (New York, NY, USA). Targeted gene sequencing was conducted at Molecular Vision Laboratory (Hillsboro, OR), or University of Utah Genome Center (Salt Lake City, UT, USA). Retinal dystrophy panels were conducted at Blueprint Genetics (Helsinki, Finland, USA), Casey Eye Institute Diagnostic Laboratory at Oregon Health & Science University (Portland, OR, USA), Prevention Genetics (Marshfield, WI, USA), or GeneDx (Gaithersburg, MD, USA). WGS was performed at New York Genome Center (New York, NY, USA). Array CGH was analyzed at Molecular Vision Laboratory (Hillsboro, OR, USA). Technical information for each gene testing is found in Table 1.

**Table 1:** Technical information of whole exome sequencing (WES) and each subsequent genetic testing for the detection of missed variants

Gene testing		Location		Technical Information^a^
Whole exome sequencing		Personalized Genomic Medicine Laboratory, Columbia University Irving Medical Center		Agilent SureSelectXT Human All Exon V5 + UTRs capture and Illumina HiSeq2500 sequencing technology was used to obtain the whole exome sequence. Analysis was performed using NextGENe software (Softgenetics) and our own proprietary analytical pipeline with 100x coverage of targeted regions; minimum 95% of region of interest covered at least 15x
Missed Variant	Gene testing	Location	Test	Technical Information^a^
MAK Alu ins (Case 18)	Targeted gene sequencing	Molecular Vision Laboratory	MAK mutation analysis	PCR amplification and Sanger sequencing for mutations in the *MAK* gene; all exons and exon/intron boundaries were sequenced
ABCA4 deep intronic (Case 23)		Molecular Vision Laboratory	ABCA4 mutation analysis	PCR amplification and Sanger sequencing for mutations in the *ABCA4* gene; all exons and exon/intron boundaries were sequenced
RPGR ORF15 (Cases 1–15)		University of Utah Genome Center	Direct sequencing of ORF15 RPGR	PCR amplification and Sanger sequencing for mutations in the *RPGR* gene
MAK Alu ins (Cases 16, 17)	Gene panel	Blueprint Genetics	Retinal dystrophy panel plus	266 genes, 8296 exons, 943,718 bases with coverage >15x, median coverage 417; 99.9% above coverage >15x; Del/Dup (CNV) analysis for known pathogenic CNVs
MAK Alu ins (Cases 19, 20)		Casey Eye Institute Diagnostic Laboratory at Oregon Health & Science University	NGS retinal dystrophy Panel (132 genes)	PCR amplification and NGS followed by Sanger sequencing of genes known to cause retinal dystrophy. All exons and exon/intron boundaries were sequenced
ABCA4 deep intronic (Case 24)		Prevention Genetics	IRD NGS sequencing panel (31 genes)	Pipeline: Titanium version 1.0.5. (average NGS coverage 528x) Titanium2 version 1.0.5. (average NGS coverage 538x). Each with 100% fraction bases covered with NS and after Sanger Backfill
PRPF31 exonic deletion CNV (Case 22)		GeneDx	Retinal dystrophy Xpanded gene panel (880 genes)	Inhouse system used to capture exonic regions and flanking splice junctions of genome. NGS on Illumina sequencing was used to sequence 100 bp or greater paired-end reads. Xome analyzer used to align the reads to hg19
GUCY2D deep intronic (Case 25)	Whole genome sequencing	New York Genome Center	WGS for undiagnosed disease	KAPA Hyper Prep kit was used to extract genomic DNA. WGS was performed on Illumina HiSeqX instrument (Illumina, CA) with 150 bp paired-end reads, minimum 30x mean coverage, minimum 85% bases to minimum 20x coverage
EYS exonic deletion CNV (Case 21)	Array CGH	Molecular Vision Laboratory	Array CGH analysis of retinal dystrophy genes	Extracted DNA was analyzed using an array CGH from OGT (Eye gene array v2). Array data was analyzed by using OGT software CytoSure

Each sequence was mapped to GRCh 37/hg19 reference sequence and analyzed using each company’s own proprietary analytical pipeline

^a^Technical information was available from the molecular genetic reports released from each sequencing company

The molecular test report of each patient was reviewed for genes known to cause IRDs. We used a joint consensus recommendation of the ACMG and the Association for Molecular Pathology [15] for the interpretation of the genetic reports. The impact of previously unreported intronic variants were predicted by using Transcript inferred Pathogenicity Score (TraP) and Human Splicing Finder bioinformatic tools. The cases with genes harboring variants that did not match the clinical phenotype were excluded.

## Results

Of 250 patients and family members that received WES between 2013 and 2018, 108 received results that reported no pathogenic variants and therefore offered no genetic explanation for their clinical diagnosis. Of these, a total of 26 cases (21 patients and 5 family members) received additional genetic testing. The remaining 82 cases did not receive subsequent genetic sequencing. WES did not identify 26 variants in the following genes: *RPGR*, *MAK*, *EYS, PRPF31*, *ABCA4*, and *GUCY2D* (Table 2). These genes are known to cause: X-linked RP (*RPGR*), autosomal recessive RP (*MAK* and *EYS*), autosomal dominant RP (*PRPF31*), Stargardt disease (*ABCA4*), and Leber congenital amaurosis (*GUCY2D*). Molecular genetic testing predicted the variants were genetically deleterious according to the ACMG guidelines. There were seven previously undescribed variants: two protein-truncating variants of *RPGR* open reading frame of exon 15 (ORF15) c.2752G > T (p.Glu918*) and *RPGR* ORF15 c.2501_2502del (p.Glu834Glyfs*244), two large *EYS* exonic deletions from exon 15 to 18 and 20 to 22, one large *PRPF31* exonic deletion from exon 1 to 9, two deep intronic variants of *ABCA4* c.4539 + 2085G > A, and *GUCY2D* c.1378 + 151C > G.

**Table 2:** Characterization of the genetic variants of inherited retinal diseases of the negative WES cases. XLRP = X-linked RP, NA = not applicable. * = premature termination of translation

Case	Age	Sex	Gene (Phenotype)	Chromosome:Genomic variant	Exon	DNA change	Protein change	Zygosity	Variant type not covered by WES
1	13	M	RPGR (XLRP)	X:38145846_38145847delCT	ORF15	c.2405_2406del	p.Glu802Glyfs*32	Hemizygous	Frameshift deletion
2	25	F	RPGR (XLRP)	X:38145846_38145847delCT	ORF15	c.2405_2406del	p.Glu802Glyfs*32	Heterozygous	Frameshift deletion
3	61	M	RPGR (XLRP)	X:38145846_38145847delCT	ORF15	c.2405_2406del	p.Glu802Glyfs*32	Hemizygous	Frameshift deletion
4	37	M	RPGR (XLRP)	X:38146058delC	ORF15	c.2194del	p.Glu732Argfs*83	Hemizygous	Frameshift deletion
5	47	M	RPGR (XLRP)	X:38145825delCT	ORF15	c.2426_2427del	p.Glu809Glyfs*25	Hemizygous	Frameshift deletion
6	49	M	RPGR (XLRP)	X:38145825delCT	ORF15	c.2426_2427del	p.Glu809Glyfs*25	Hemizygous	Frameshift deletion
7	41	M	RPGR (XLRP)	X:38145224delCC	ORF15	c.3027_3028del	p.Glu1010Glyfs*68	Hemizygous	Frameshift deletion
8	74	M	RPGR (XLRP)	X:38145775del38145775	ORF15	c.2467_2477del	p.Arg826Glyfs*8	Hemizygous	Frameshift deletion
9	55	M	RPGR (XLRP)	X:38145775delCTCT	ORF15	c.2474_2477del	p.Glu825Glyfs*263	Hemizygous	Frameshift deletion
10	21	M	RPGR (XLRP)	X:38145500C > A	ORF15	c.2752G > T	p.Glu918*	Hemizygous	Nonsense
11	44	M	RPGR (XLRP)	X:38145182C > A	ORF15	c.3070G > T	p.Glu1024*	Hemizygous	Nonsense
12	74	M	RPGR (XLRP)	X:38145182C > A	ORF15	c.3070G > T	p.Glu1024*	Heterozygous	Nonsense
13	44	M	RPGR (XLRP)	X:38145846_38145847delCT	ORF15	c.2405_2406del	p.Glu802Glyfs*32	Hemizygous	Frameshift deletion
14	19	F	RPGR (XLRP)	X:38145846_38145847delCT	ORF15	c.2405_2406del	p.Glu802Glyfs*32	Heterozygous	Frameshift deletion
15	39	M	RPGR (XLRP)	X:38145750_38145751delCT	ORF15	c.2501_2502del	p.Glu834Glyfs*244	Hemizygous	Frameshift deletion
16	35	M	MAK (RP62)	6:10791926_10791927ins(353)	10 of 14	c.1297_1298ins(353)	p.Lys433_Lys434ins (1)	Homozygous	353-bp Alu repeat insertion
17	33	M	MAK (RP62)	6:10791926_10791927ins(353)	10 of 14	c.1297_1298ins(353)	p.Lys433_Lys434ins (1)	Homozygous	353-bp Alu repeat insertion
18	57	M	MAK (RP62)	6:10791926_10791927ins(353)	10 of 14	c.1297_1298ins(353)	p.Lys433_Lys434ins (1)	Homozygous	353-bp Alu repeat insertion
19	76	M	MAK (RP62)	6:10791926_10791927ins(353)	10 of 14	c.1297_1298ins(353)	p.Lys433_Lys434ins (1)	Homozygous	353-bp Alu repeat insertion
20	45	M	MAK (RP62)	6:10791926_10791927ins(353)	10 of 14	c.1297_1298ins(353)	p.Lys433_Lys434ins (1)	Homozygous	353-bp Alu repeat insertion
21	51	F	EYS (RP25)	6:65603049_65657244del 6:65506901_65555979del	15–18 of 43 20–22 of 43	NA NA	NA NA	Heterozygous Heterozygous	Deletion Deletion
22	40	M	PRPF31 (RP11)	19:54577171_54630008del	1–10 of 14	NA	NA	Heterozygous	Copy number loss
23	43	F	ABCA4 (STGD1)	1:94525509 T > C 1:94473807C > T	Intron 30 of 49 42 of 50	c.2160 + 584A > G c.5882G > A	NA p.Gly1961Glu	Heterozygous Heterozygous	Intronic Missense
24	76	M	ABCA4 (STGD1)	1:94492916C > T 1:94544977A > T	Intron 30 of 49 9 of 50	c.4539 + 2085G > A c.1140 T > A	NA p.Asn380Lys	Heterozygous Heterozygous	Intronic Missense
25	6	F	GUCY2D (LCA1)	17:7906676CTT > CTTTT 17:7910183G > C	2 of 20 Intron 4 of 19	c. 312_313dupTT c.1378 + 151C > G	p.Cys105Phefs*25 NA	Heterozygous Heterozygous	Frameshift insertion Intronic

**Fig. 1:**
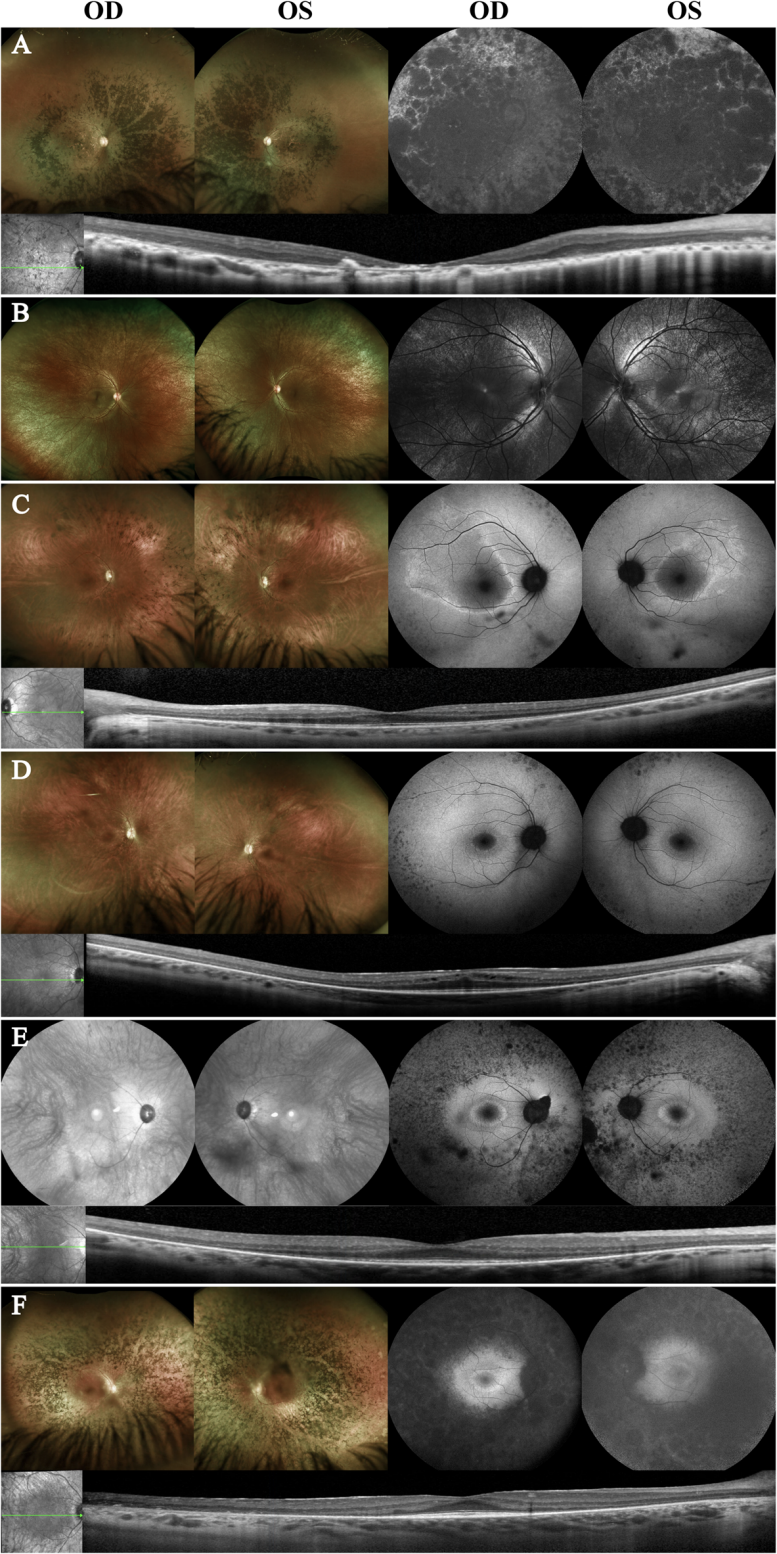
Images of selected cases of retinitis pigmentosa: *RPGR* (**a** and ***b***), *MAK* (**c** and **d**), *EYS* (**e**), and *PRPF31* (**f**). Color fundus photography (left panels), short-wave fundus autofluorescence imaging (SW-FAF, right panels), and spectral-domain optical coherence tomography scans (SD-OCT, bottom panels) were performed. Blue reflectance imaging (488 nm, excitation) of Case 14 displayed the tapetal-reflex, a radiating pattern of hyperreflectivity commonly observed in *RPGR* carriers (B, right panel). The observed characteristic findings of retinitis pigmentosa include bilateral widespread intraretinal pigmentation, hyperautofluorescent rings on the macula, and shortened or absent EZ line. No color fundus photography was performed for Case 21; infrared reflectance imaging was performed instead (E, left panel)

**Fig. 2:**
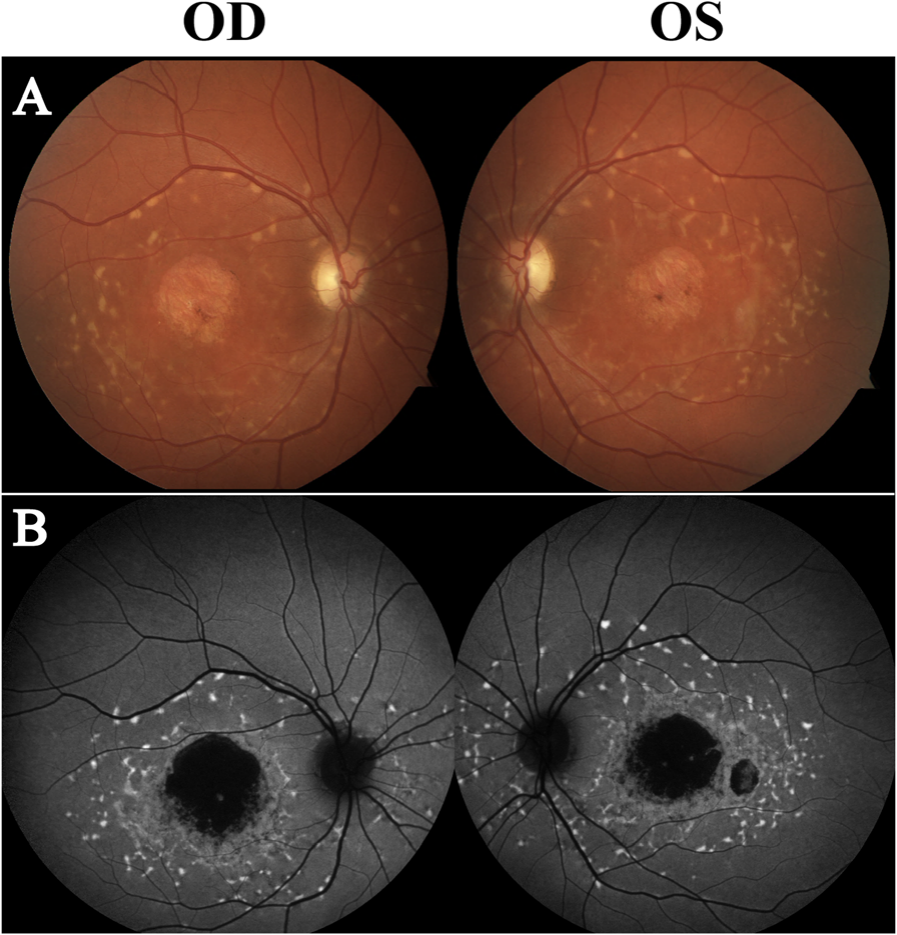
Color fundus photography (top panels) and short-wavelength fundus autofluorescence (SW-FAF, bottom panels) of selected cases of Stargardt disease (**a** and **b**, Case 23). Case 23 presented with peripapillary-sparing and yellow-white pisciform lesions that are characteristic of Stargardt disease (**a**). The lesions are observed as hyperautofluorescent flecks on SW-FAF (**b**)

Overall, WES did not detect 15 *RPGR* variants found in ORF15, including 12 frameshift deletions and three nonsense mutations. These variants were identified by targeted gene sequencing. The homozygous 353-bp Alu insertion variant in exon 9 of the *MAK* gene was also missed by WES, which was identified by a gene panel (Retinal Dystrophy Panel Plus, Blueprint Genetics). In the *EYS* gene, WES did not detect two large exonic deletion variants spanning exons 15 to 18 and 20 to 22 out of a total of 43 exons, each over 54 kb and 49 kb in length, respectively. These were subsequently identified with array CGH of IRD genes. The exonic deletion variant of over 52 kb in length in the *PRPF31* gene that spanned exons 1 to 9 out of a total of 14 exons was identified by a gene panel (Retinal Dystrophy Xpanded Test of 880 genes, GeneDx). In the *ABCA4* gene, WES did not identify two deep intronic variants, c.4539 + 2085G > A and c.2160 + 584A > G, which were discovered by targeted gene sequencing of the *ABCA4* gene. The deep intronic variant c.1378 + 151C > G in the *GUCY2D* gene that was not identified by multiple tests, including WES, array CGH analysis, and single-gene analysis for deletion and duplication, was subsequently detected by WGS. Clinical descriptions of selected cases representative of each gene are provided below. The case images of RP are shown in Fig. 1, and those from STGD are shown in Fig. 2. Fundus photography could not be taken for Case 25 due to body-rocking behavior, which is a manneristic behavior of children with visual impairment [16].

### RPGR

Case 13 is a 44-year old man who was diagnosed with RP at the age of 8 (Fig. 1a). He began to notice vision changes at the age of 18 that worsened by the age of 21. On presentation, best-corrected visual acuity (BCVA) was count fingers at 2 feet bilaterally. On fundoscopy, dense intraretinal pigment migration was observed throughout the periphery. Wide-spread retinal atrophy could also be appreciated. SW-FAF imaging revealed hypoautofluorescence throughout the posterior pole, suggestive of widespread retinal pigment epithelium (RPE) atrophy. SD-OCT scans showed an absence of the outer retinal layers along with increased signal transmittance of the choroid. Fundus ophthalmic examination of his daughter, Case 14, revealed a radiating pattern of hyperreflectivity that manifests as patchy radial streaks on fundoscopy, referred to as the tapetal-like reflex, a characteristic phenotype commonly observed in *RPGR* carriers (Fig. 1b) [17, 18]. Targeted sequencing of the *RPGR* gene detected the heterozygous c.2405_2406delAG (p.Glu802Glyfs*32) variant in the proband and his daughter.

### MAK

Case 16 is a 35-year old man of Ashkenazi Jewish descent who was diagnosed with RP at the age of 33 (Fig. 1c). He was referred to our clinic for genetic counseling. BCVA was 20/20 and 20/25 for the right and left eye, respectively. On fundoscopy, intraretinal pigment migration was observed bilaterally, with increased concentration at the nasal aspect. SW-FAF revealed a hyperautofluorescent ring on each eye, with irregular borders on the superior-temporal aspect of the ring. SD-OCT scans revealed retinal thinning and the absence of the ellipsoid zone (EZ) line in the periphery, while the retinal layers and EZ line were conserved centrally on the macular area. A gene panel (Retinal Dystrophy Panel Plus, Blueprint Genetics) identified the homozygous c.1297_1298insAlu (p.Lys433insAlu) variant for Case 16 and his brother, Case 17. Fundoscopy of Case 17 revealed small spots of intraretinal pigment migration in the inferior nasal region (Fig. 1d). FAF showed hyperautofluorescent rings with regular borders on each eye. SD-OCT scans showed same features as the proband’s OCT images.

### EYS

Case 21 is a 51-year-old woman who was diagnosed with RP 20 years ago (Fig. 1e). On presentation, she reported a continuous reduction of night vision and peripheral vision. BCVA was 20/25 bilaterally. SW-FAF revealed a hyperautofluorescent ring on the macula and intraretinal pigment migration in the periphery. SD-OCT scans revealed retinal thinning and absence of the EZ line on the periphery, while the retinal layers and EZ line were conserved centrally on the macular area. Array CGH of IRD genes identified two heterozygous exonic deletions in the *EYS* gene (exon 15 to 18 and exon 20 to 22).

### PRPF31

Case 22 is a 40-year-old man who presented with BCVA of 20/40 bilaterally (Fig. 1f). The patient’s family history was significant for multiple members affected by RP: his sister, father, two paternal aunts, and paternal grandmother. Fundoscopy revealed widespread, dense intraretinal pigment migration throughout the periphery, indicating retinal atrophy. SW-FAF imaging revealed hypoautofluorescence on the periphery, with a hyperautofluorescent ring on the macula. On SD-OCT scans, peripheral retina thinning was observed, with conservation of the retina layers, including the EZ line, on the macular area. A gene panel (Retinal Dystrophy Xpanded Test of 880 genes, GeneDx) identified the heterozygous deletion of exons 1 to 9 in the *PRPF31* gene.

### ABCA4

Case 23 is a 43-year old woman diagnosed with Stargardt disease at the age of 18 when she experienced an onset of central vision problems (Fig. 2a). BCVA was 20/200 bilaterally. There was no history of similar vision problems in her family. Fundoscopy revealed an atrophic macula, with pisciform, yellow-white flecks surrounding the macula. On SW-FAF, dense hypoautofluorescence was observed in the macular area, indicative of RPE atrophy. Hyperautofluorescent flecks were also observed on the posterior pole. WES identified a heterozygous c.5882G > A (p.Gly1961Glu) variant, but a second variant was not detected. Given the autosomal recessive nature of the disease, further targeted sequencing of the *ABCA4* gene identified a second heterozygous c.2160 + 584A > G intronic variant in the same gene, consistent with the clinical diagnosis.

### GUCY2D

Case 25 is a 6-year-old girl attending school for the visually impaired. Nystagmus was first noted at the age of 3 months and congenital blindness was confirmed at 9 months. LCA was diagnosed before 1 year of age based on her clinical history and ffERG results. BCVA was light perception, bilaterally. On fundoscopy, mild arterial attenuation at the peripheral retina was noted, and a ffERG performed under anesthesia revealed extinguished cone and rod responses, which was consistent with the clinical diagnosis of LCA. WES identified a heterozygous c.312_313dupTT (p.Cys105Phefs*25) variant in the *GUCY2D* gene, however, this finding could not explain the recessive phenotype. WGS identified the second heterozygous c.1378 + 151C > G intronic variant in the *GUCY2D*, consistent with the clinical diagnosis.

## Discussion

WES has contributed to a significant advancement in our understanding of the genetic causes of inherited diseases through the discovery of novel variants, enhancement of important genotype-phenotype associations, and progression of gene-directed therapy. Approximately 2600 gene therapy clinical trials in 38 countries have been or are being conducted [19].

WES as first-tier genetic testing enabled an unbiased search for the genetic causes of disease. This “WES-first” approach has been associated with a lower incremental cost per additional diagnosis than the traditional WES-later approach [20–24]. The cost of WES has continuously declined to a close equivalent to those of targeted or panel sequencing, which discourages the notion of performing WES after targeted or panel sequencing. The WES-first approach curtails the number of genetic testing and the associated financial burden on patients, which are a significant barrier to testing [25]. A similar downward trend is observed for the cost of WGS, which further encourages the selection of NGS over Sanger sequencing used for targeted or panel sequencing.

We categorized the limitations of WES into two classes, based on whether the missed variants were located within or beyond the sequencing scope (Table 3). The first class of limitations includes structural variations such as GA-repetitive sequence and CNVs. *RPGR* ORF15, which constitutes a large 3′ terminal region of the *RPGR* gene, is a mutational hotspot associated with up to 60% of pathogenic mutations of X-linked RP [26]. In our cohort, *RPGR* ORF15 variants were the most common, as observed in Cases 1 to 15. Compared to the constitutive RPGR isoform that spans exons 1 to 19, the ORF15 isoform terminates in intron 15, a GA-rich region that encodes Glu-Gly acidic domains [26]. GA-rich regions, as with long repeats of other di- and trinucleotides, act as a primary algorithmic challenge in sequence assembling, as the sequence reads lack the capacity to span long repetitive elements [27, 28]. Consistently, failures to assemble these structures have been attributable to the gaps in the human genome [29–31]. Characteristic fundus features of RP, such as peripheral intraretinal pigment migration and a hyperautofluorescent ring on the macula, and significant history such as nyctalopia, X-linked mode of inheritance, and severe disease at a relatively young age formed the basis for requesting targeted sequencing of the *RPGR* gene following the negative WES analysis. Additionally, the tapetal-like reflex observed in the daughter strongly suggested a carrier status for an *RPGR* variant (Fig. 1b).

**Table 3:** Classes of variants unidentified by WES

	Gene Mutation	Summary of Underlying Reason	Follow-up Analysis
Structural Variations	*RPGR* ORF15 variants	High GA % regions	Targeted gene sequencing
	*MAK* 353-bp Alu insertion	Platform	Retinal dystrophy gene panel plus or targeted gene sequencing using the ABI sequencer
	*EYS* exonic deletions	CNV	Array CGH of IRD genes
	*PRPF31* exonic deletion	CNV	Retinal dystrophy Xpanded gene panel
Deep Intronic Variations	*ABCA4* c.4539 + 2085G > A	Past immediate introns	IRD gene panel
	*ABCA4* c.2160 + 584A > G	Past immediate introns	Targeted gene sequencing
	*GUCY2D* c.1378 + 151C > G	Past immediate introns	WGS

*CNV* copy number variation; *CGH* comparative gene hybridization; *IRD* inherited retinal disease; *WGS* whole genome sequencing

**Fig. 3:**
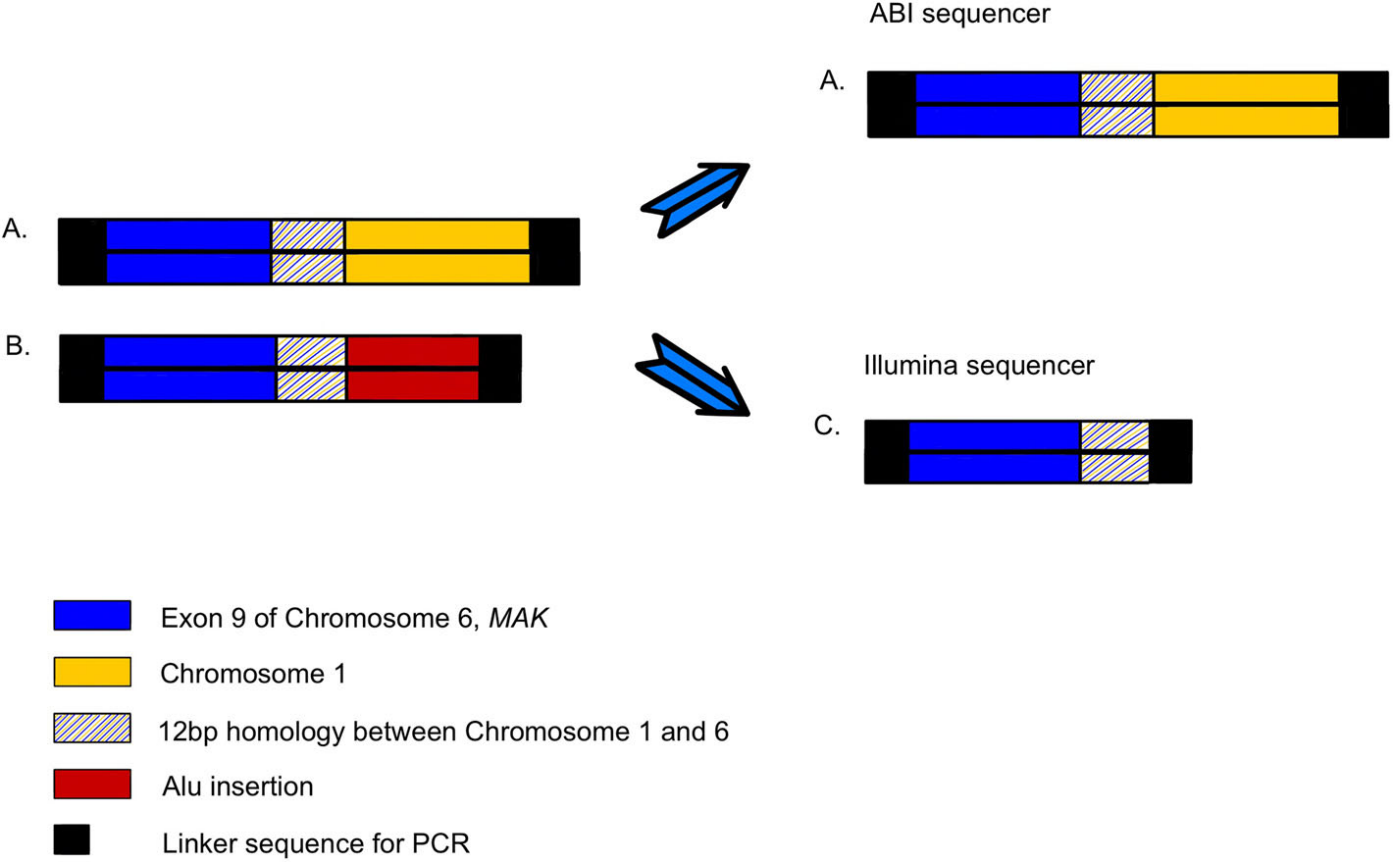
DNA fragment present at Exome capture. The library contains a chimeric fragment (**a**) and the proband’s fragment containing the Alu insertion (**b**). With ABI sequencing, genomic fragments containing the Alu-MAK junction were removed. The removal of these fragments led to the paradoxical detection of the mutation. With Illumina sequencing, these Ala-MAK junction fragments were not completely removed. Subsequently, the Ala-MAK junction was excised, creating fragment **C**, which is similar to the wild-type fragment and thus the mutation was not detected

The homozygous 353-bp Alu insertion in exon 9 of the *MAK* gene is a common variant found in the Ashkenazi Jewish population, occurring at a frequency of 1 in 55 [32]. It is predicted to generate 31 incorrect amino acids leading to protein truncation. The nasal pigmentation, characteristic of *MAK*-associated disease (Fig. 1c) [33], and the patient’s Ashkenazi Jewish background increased the likelihood of the *MAK* variant, prompting analysis using an additional retinal dystrophies panel following the negative WES report. In a previous study by Tucker et al., the variant was successfully identified by WES using the Applied Biosystems sequencing platform (ABI, SOLiD 4hq) [32]. They proposed a mechanism to explain the failure of variant calling by WES that uses the Illumina HiSeq sequencing platform, which is used in our hospital. It suggested that a chimeric DNA molecule was introduced into the sequencing library, composed of chromosome 1, 12-bp homology between chromosome 1 and 6, and exon 9 of chromosome 6 containing the *MAK* gene (Fig. 3a). Before exome capture, the ABI sequencer had physically removed the proband’s Alu-insertion *MAK* sequence (Fig. 3b). Therefore, the chimeric DNA fragment was captured instead, and interpreted as a compound heterozygous mutation. In contrast, the Illumina sequencer targeted and excised the proband’s Alu-insertion, producing the proband’s DNA fragment with only exon 9 (Fig. 3c). Consequently, the excision by the genome analysis toolkit allowed the proband’s DNA fragment to masquerade as a normal *MAK* sequence and thus led to a negative variant calling. The discrepancy in performance between different WES sequencing platforms attests to the technical limitation of the method and reduces its reliability.

Three exonic deletion variants were not detected by WES: two in the *EYS* gene and one in the *PRPF31* gene. The WES pipeline is prone to miss these variant types because it is constructed to detect SNVs or short indels [34]. In a study of 384 Mendelian disease genes, between 4.7 and 35% of pathogenic variants were CNVs, indicating that complementing WES with CNV analysis, such as multiplex ligation-dependent probe amplification (MLPA) or an array based approach, enhances the clinical sensitivity of the genetic testing [35].

The second class of limitations of WES involves the remaining 98% of the genome beyond its sequencing scope. By design, WES does not cover intronic variants, as exons have been perceived as the primary regions of the genome that when disrupted are responsible for causing disease. However, genome sequencing has revealed the clinical significance of structural and regulatory variants of the noncoding genome. Deep intronic mutations can be pathogenic by activating non-canonical splice sites, changing splicing regulatory elements, or disrupting transcription regulatory motifs [36].

Three intronic missense variants were not identified by WES: two in *ABCA4*, and one in the *GUCY2D* gene. The genetic variants of deep intronic nature in the *ABCA4* gene have been previously reported as the cause for the missing variant of STGD1 [37]; 67% of 36 cases with undetected variants from exome sequencing were resolved with the finding of deep intronic variants and 17 variants were predicted to have deleterious effects. Therefore, we predict a high likelihood that the deep intronic variants found in our cohort share the same mechanisms of disease as those reported; namely, the insertion of pseudoexons as well as activation and disruption of exonic splice enhancer elements [36, 37]. The intronic variant of Case 23 is likely to be pathogenic based on the predicted TraP score of 0.625 and its effect of causing donor site breakage as predicted by Human Splicing Finder. This is consistent with the observation by Zernant et al. on the positive disease association of the variant due to the creation of a new donor site and the predicted conservation of the region in primates [38]. Conversely, the deep intronic variant found in Case 24 is a variant that has previously not been reported and associated with disease. It is predicted by Human Splice Factor to cause an alteration of an intronic exon splicing silencer (ESS) site with a TraP score of 0, supporting its status as a variant of uncertain significance. The remaining missense ABCA4 variant harbored in Case 24 is also a variant of uncertain significance, rendering the case unresolved with no identified pathogenic variant. Both targeted gene sequencing and WES could not identify the pathogenic variant, making WGS as a fitting candidate sequencing platform to provide the most comprehensive search for the cause of disease.

WGS detected the heterozygous deep intronic variant in the *GUCY2D* gene harbored in Case 25, which is predicted to activate a new splicing donor site. Like STGD, LCA shows autosomal recessive inheritance that manifests with the presence of bi-allelic variants. Therefore, when WES identifies only one variant in a gene known to cause LCA, it justifies for the subsequent search for the second variant, most likely one of a deep intronic nature, as this type is commonly associated with LCA. Previous studies have consistently established the association of a deep intronic c.2991 + 1655A > G variant in the *CEP290* gene with LCA, occurring in more than half of *CEP290*-associated cases [39, 40]. This common variant correlates with the severe congenital retinal phenotype of LCA, resulting in legal blindness at a young age [41]. Therefore, when WES identifies one variant and a second variant is expected within the gene, Sanger sequencing of the suspected intronic region(s) may be more economical. Alternatively, WES may be customized to include common intronic regions of a specific gene that were previously reported, like that of *CEP290* c.2991 + 1655A > G. If the search warrants an unbiased approach, WGS would be recommended.

Our study illustrates that following a negative WES report, further genetic testing, such as targeted gene panels that cover deep intronic and highly repetitive regions or WGS, is needed to account for these limitations. These alternative tests are particularly important when the patient’s clinical phenotype is compelling. However, the interpretive limitation of these sequencing platforms should also be noted. The clinical significance of the identified variant is predicted based on previously reported findings, which constitute a body of medical knowledge that is continuously expanding.

Further investigation of gene variants in a larger cohort will strengthen the need to re-evaluate negative WES results with additional genetic testing. Although it functions with a lower overall coverage depth of 30x compared to WES (100x), WGS performs at a higher hybridization efficiency because it has a more consistent read depth and covers the non-targeted regions of WES. Compared to using WES alone, supplementing unresolved WES cases with WGS identified 14 out of 45 additional pathogenic variants, which translates to a detection rate of 31% [14]. However, the *RPGR* ORF 15 region still represent a technical challenge for WGS because of the highly repetitive regions that lead to poor coverage. Further analysis, including targeted long-range PCR following DNA fragmentation and long read high depth sequencing, are therefore required in addition to WES, or WGS are required for these types of cases.

## Conclusions

Despite the high diagnostic yield of WES, there are inherent technical limitations that lead to missed variant callings. As achieving genetic diagnosis is imperative for clinicians and patients to move forward with potential treatments such as gene replacement therapy, a negative WES analysis should be re-evaluated when compelling clinical findings support the presentation of a distinct genetic etiology. We used 14 targeted gene sequencing, 10 gene panels, one WGS, and one array CGH to identify the undetected gene variants of high GA-repeat regions of *RPGR* ORF15, *MAK* 353-bp Alu insertion, large exonic deletions in *EYS* and *PRPF31*, and intronic variants in *ABCA4* and *GUCY2D*. While the current cost per diagnosis is higher for WGS compared to that of WES, it continues to fall [14], encouraging an increased utilization of WGS in the clinic setting. We predict that WGS will successfully identify many of the variants observed in this study due to its genome-wide scope of sequencing to detect deep intronic variants, and increased power to identify structural genomic variants such as DNA rearrangements and large CNVs [14]. Furthermore, we emphasize the need for the continued discovery of novel variants in order to ultimately overcome the current limit in medical knowledge of genes known to cause IRDs.

## Data Availability

All data supporting the results reported in this study are available from the corresponding author upon request.

## Abbreviations

CGH: Array comparative genomic hybridization
CNVs: Copy number variations
IRDs: Inherited retinal diseases
LCA: Leber congenital amaurosis
NGS: Next generation sequencing
RP: Retinitis pigmentosa
STGD: Stargardt disease
WES: Whole exome sequencing
WGS: Whole genome sequencing
